# Complete Mitochondrial Genome of a Gymnosperm, Sitka Spruce (*Picea sitchensis*), Indicates a Complex Physical Structure

**DOI:** 10.1093/gbe/evaa108

**Published:** 2020-05-25

**Authors:** Shaun D Jackman, Lauren Coombe, René L Warren, Heather Kirk, Eva Trinh, Tina MacLeod, Stephen Pleasance, Pawan Pandoh, Yongjun Zhao, Robin J Coope, Jean Bousquet, Joerg Bohlmann, Steven J M Jones, Inanc Birol

**Affiliations:** e1 Genome Sciences Centre, BC Cancer, Vancouver, British Columbia, Canada; e2 Forest Genomics, Institute for Systems and Integrative Biology, Université Laval, Quebec, Quebec, Canada; e3 Michael Smith Laboratories, University of British Columbia, Vancouver, British Columbia, Canada

**Keywords:** gymnosperms, Sitka spruce, organelle, genome assembly, sequencing, ABySS

## Abstract

Plant mitochondrial genomes vary widely in size. Although many plant mitochondrial genomes have been sequenced and assembled, the vast majority are of angiosperms, and few are of gymnosperms. Most plant mitochondrial genomes are smaller than a megabase, with a few notable exceptions. We have sequenced and assembled the complete 5.5-Mb mitochondrial genome of Sitka spruce (*Picea sitchensis*), to date, one of the largest mitochondrial genomes of a gymnosperm. We sequenced the whole genome using Oxford Nanopore MinION, and then identified contigs of mitochondrial origin assembled from these long reads based on sequence homology to the white spruce mitochondrial genome. The assembly graph shows a multipartite genome structure, composed of one smaller 168-kb circular segment of DNA, and a larger 5.4-Mb single component with a branching structure. The assembly graph gives insight into a putative complex physical genome structure, and its branching points may represent active sites of recombination.

## Introduction

Plant mitochondrial genomes are amazingly diverse and complex (Mower et al. 2012). Land plant mitochondrial genomes range in size from 66 kilobases (kb) for the parasitic angiosperm *Viscum scurruloideum* (Skippington et al. 2015) to >11 megabases (Mb) in the case of the flowering plant *Silene conica* (Sloan et al. 2012). Although their genome structure is often portrayed as a circle, the true physical structure of their genome appears to be a variety of circles, linear molecules, and complex branching structures (Backert et al. 1997; Backert and Börner 2000). Although many species have a single master circle representation of their mitochondrial genome, others are composed of more than a hundred circular chromosomes (Sloan et al. 2012). The precise mechanism of how plant mitochondria replicate and maintain their DNA is not yet fully understood (Cupp and Nielsen 2014). It is hypothesized that recombination-dependent replication plays a role, giving a functional role to the repeat sequences often observed in mitochondria (Gualberto et al. 2014). This model does not fully explain how genomic copy number is regulated and maintained (Oldenburg and Bendich 2015), particularly in multipartite genomes (Vlcek et al. 2011). Although angiosperm mitochondrial genomes are well studied with numerous complete genomes available, gymnosperm mitochondrial genomes were scarcer until recently: One from each of the cycads (Chaw et al. 2008), ginkgos, gnetales (Guo et al. 2016), and conifers (Jackman et al. 2016). This year saw a number of mitochondrial genomes published in short succession (Guo et al. 2020; Kan et al. 2020; Sullivan et al. 2020). Although other gymnosperm mitochondrial genomes are smaller than a megabase, conifer mitochondrial genomes can exceed five megabases (Jackman et al. 2016), larger than many bacteria. Compared with other plants, conifers also tend to have very large nuclear genomes (De La Torre et al. 2014), particularly the spruce species (Birol et al. 2013; Nystedt et al. 2013; Warren et al. 2015). As plant mitochondrial genomes typically have <100 genes, what role this expanse of DNA serves, if any, remains mysterious. 

Assembling plant mitochondrial genomes is difficult due to the presence of large (up to 30 kb) perfect repeats, which may be involved in active recombination, and hypothesized recombination-dependent replication (Gualberto et al. 2014). A hybrid assembly of both long reads, which are able to span most repeats, and accurate short sequencing reads, which correct indel errors, is well suited to tackle these challenging genome features. Hybrid assembly of long and short reads has been applied to assemble the plastid genome of *Eucalyptus pauciflora* (Wang et al. 2018), as well as plant mitochondrial genomes (Kovar et al. 2018; Kozik et al. 2019).

Annotating plant mitochondrial genomes is also challenging, due to numerous features of plant mitochondria that are not typical of most organisms. For one, RNA editing of C-to-U is pervasive, and this process creates AUG start codons by editing ACG to AUG (Hiesel et al. 1989) or by editing GCG to GUG, an alternative start codon used by some plant mitochondrial genes (Sakamoto et al. 1997). RNA editing can also create stop codons in a similar fashion. Further complicating annotation using available bioinformatics pipelines, the typical GU-AG splice site expected by most splice-aware alignments tools is instead GNGCG-AY (Y denotes C or T) for group II introns (Lambowitz and Zimmerly 2011). Also, *trans*-spliced genes are common in mitochondrial genomes (Kamikawa et al. 2016; Guo et al. 2020), and no purpose-built software tool exists for identifying and annotating *trans*-spliced genes. To add further difficulty, *trans*-spliced exons may be as small as 22 bp, as is *nad5* exon 3 of gymnosperms (Guo et al. 2016) and other vascular plant mitochondria (Knoop et al. 1991). For these reasons, annotating a plant mitochondrial genome remains a laborious and manual task.

In this study, we report on the sequencing, assembly and annotation of the mitochondrial genome of Sitka spruce (*Picea sitchensis*, Pinaceae), a widely distributed conifer in the coastal regions of the Pacific Northwest of North America. We show that this mitochondrial genome is one of the largest among plants and exhibits a multipartite genome structure.

## Materials and Methods

### Genome Sequencing and Assembly

Genomic DNA was extracted from young Sitka spruce (*P. sitchensis* [Bong.] Carrière, genotype Q903) needles, as described in Coombe et al. (2016). We constructed 18 Oxford Nanopore 1D sequencing libraries, 16 by ligation of 1–7 µg of lightly needle-sheared genomic DNA and 2 by rapid transposition of 0.6 µg of unsheared genomic DNA, and sequenced these on 18 MinION R9.4 flow cells. This whole genome sequencing produced 98 Gb in 9.6 million reads (SRA accession SRX5081713), yielding 5-fold depth of coverage of the roughly 20 Gb nuclear genome, and 26-fold depth of coverage of the mitochondrial genome. We first obtained a rough but computationally efficient assembly using Miniasm (Li 2016), after trimming adapter sequences with Porechop (Wick et al. 2017a) and polished the resulting assembly with Racon (Vaser et al. 2017). We selected contigs with homology to the white spruce (*Picea glauca*, interior white spruce genotype PG29) mitochondrial genome (Jackman et al. 2016) using Bandage (Wick et al. 2015), retaining contigs with at least one ≥5-kb alignment to the white spruce mitochondrion by BlastN (Altschul et al. 1990). We then proceeded to align back the nanopore reads to our draft Sitka spruce assembly with minimap2 (Li 2018), segregate aligned reads and assemble them de novo first with Unicycler (Wick et al. 2017b), and then with Flye (Kolmogorov et al. 2019), as described (supplementary methods, Supplementary Material online). The final genome sequence was obtained by polishing with Pilon (Walker et al. 2014) using Illumina HiSeq sequencing reads of the same DNA extraction (supplementary fig. S1, Supplementary Material online).

### Annotation

We annotated coding genes and non-coding rRNA and tRNA genes using automated methods where possible, and performed manual inspection to refine these automated annotations. We used Prokka (Seemann 2014), which uses Prodigal (Hyatt et al. 2010) to identify single-exon coding genes and open reading frames (ORFs). We used MAKER (Holt and Yandell 2011), which uses BLASTP and Exonerate (Slater and Birney 2005) to identify *cis*-spliced coding genes. Prokka used ab initio predictions from Prodigal. MAKER used evidence alignments only. Genes in GenBank from *Viridiplantae* mitochondria were used as evidence. The annotations of Prokka and Maker were combined, along with the manually annotated *cis*-spliced and *trans*-spliced genes. No species-specific TE libraries were used.

We used tRNAscan-SE (Lowe and Eddy 1997) and Aragorn (Laslett 2004) to identify tRNA. We used RNAmmer (Lagesen et al. 2007) and Barrnap (Seemann 2014) to identify rRNA. We used RNAweasel (Lang et al. 2007) and Infernal (Nawrocki et al. 2009) to identify group II introns. RFAM motif Domain-V (RM00007) represents domain V of group II introns, and RFAM family Intron_gpII (RF00029) represents both domains V and VI (Kalvari et al. 2018).

## Results and Discussion

### Complete Genome Assembly

**Fig. 1.**
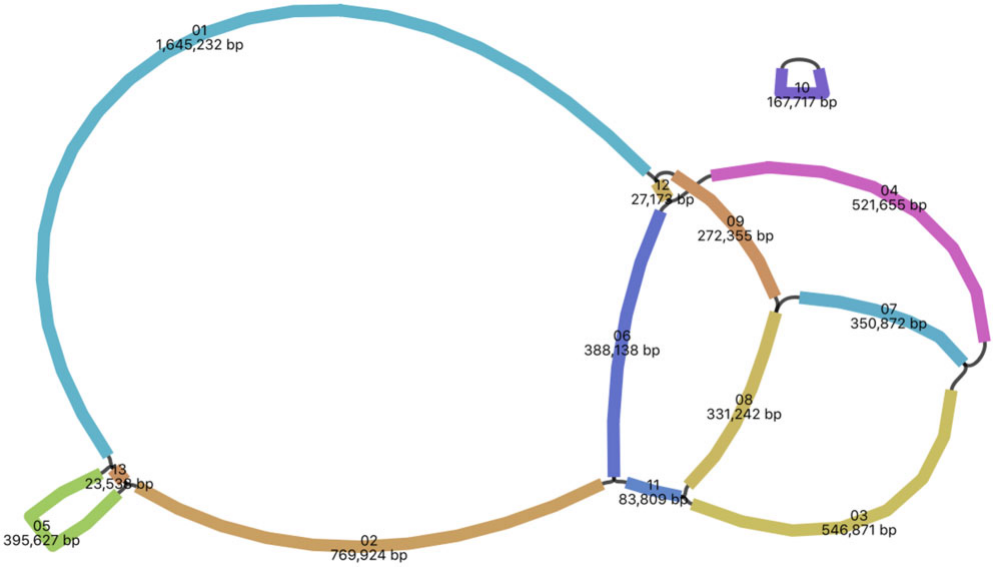
The assembly graph of the mitochondrial genome of Sitka spruce. Each colored segment is labeled with its size and named 01–13 by rank of size. Only segment 12 and 13 representations are inferred as repeats. All segment adjacencies are supported by the long reads, indicating a complex branching genomic structure.

The complete mitochondrial genome of Sitka spruce is 5.52 Mb assembled in 13 segments, and has a GC content of 44.7%. The assembly statistics are summarized in table 1. The genome assembly is composed of two components: A 168-kb circular segment (labeled 10), and a larger 5.36-Mb component composed of 12 segments, as visualized in figure 1 using Bandage (Wick et al. 2015). The eleven largest segments, ranging in size from 84 kb to 1.65 Mb, have similar depth of coverage, assumed to represent single-copy genomic segments. The two smallest segments (27 and 24 kb labeled 12 and 13 in figure 1, respectively, representing <1% of the mitochondrial genome) exhibit an estimated copy number of two based on their depth of sequencing coverage. No sequence variation is evident in these two repeats. An absence of variation in the repeat implies that they may be involved in active recombination (Maréchal and Brisson 2010). Though 10% of reads are >24 kb, no reads fully span these repeats.

**Table 1:** Summary of Assembly Statistics

Assembly Statistics	
Number of contigs	13
Assembly N50	547 kb
Undetermined bases	None
Largest contig	1.65 Mb
Total reconstruction	5.53 Mb
GC content	44.7%

The complete mitochondrial genome assembly of Sitka spruce assembled from Oxford Nanopore sequencing is composed of 13 contigs >20 kb with an N50 length of 547 kb. The use of long reads was critical in achieving this contiguity and completeness. In contrast, the draft mitochondrial genome assembly of white spruce was assembled from paired-end and mate-pair Illumina sequencing and is composed of 117 contigs >2 kb arranged in 36 scaffolds with a contig and scaffold N50 length of 102 and 369 kb, respectively (Jackman et al. 2016). The fragmented state of the white spruce mitochondrial assembly provided little information as to the structure of the mitochondrial genome, whereas the Sitka spruce assembly graph (fig. 1) suggests a multipartite genome structure.

The complete genome is composed of 1.7% (93 kb) of genes with known function, 28.0% (1,545 kb) of 6,806 ORFs (each of at least 90 bp), 3.7% (205 kb) of repeats, and 66.6% unclassified sequences. Of the ORFs, 1,039 are at least 300 bp (100 amino acids) in size and compose 7.2% (400 kb) of the genome. Aligning the ORFs with BLASTP, 63 ORFs (17 ORFs of at least 300 bp) have a significant (*E* < 0.001) hit to the nr database. Plastid-derived sequences compose 0.25% (14 kb) of the genome spread across 24 segments.

The nuclear repeats LTR/Gypsy compose 51% of the repeat sequence, and LTR/Copia compose 7%. The genome also has non-transposable element repeats; simple repeat sequences compose 34%, low complexity sequences compose 3%, and 5% are other repeat sequences. The 36-bp Bpu repeat sequence is present in roughly 500 copies in *Cycas taitungensis* and roughly 100 copies in *Ginkgo biloba* (Guo et al. 2016). We find only a single full-length copy with four mismatches in Sitka spruce, similar to *Welwitschia mirabilis*.

### Genes

**Fig. 2.**
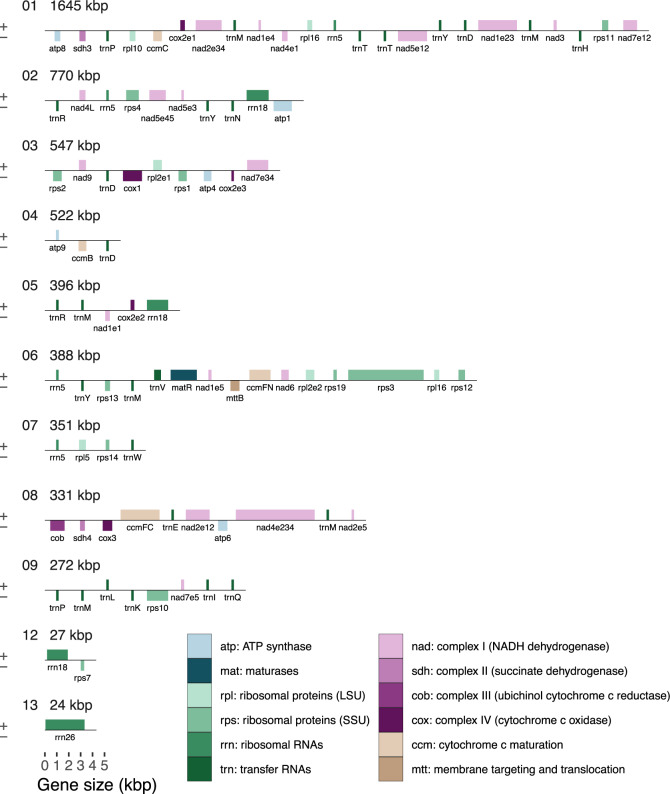
The order, orientation, and size of the genes within the Sitka spruce mitochondrial genome. Each box is proportional to the size of the gene including introns, except that genes <200 nucleotides are shown as 200 nucleotides. Intergenic regions are not to scale. Contigs 10 and 11 did not contain any annotated genes and are not shown.

The mitochondrial genome of Sitka spruce has 41 distinct protein coding genes with known function, 3 distinct rRNA genes (supplementary table S1, Supplementary Material online), and 27 distinct tRNA genes representing 18 distinct anticodons (supplementary table S2, Supplementary Material online). The relative order and orientation of these genes are shown in figure 2. The 41 known protein coding genes found in the gymnosperm mitochondria *C. taitungensis* (Chaw et al. 2008) and *G. biloba* (Guo et al. 2016) are also found in Sitka spruce. The 29 introns, 16 *cis*-spliced, and 13 *trans*-spliced, are found in 10 protein coding genes, two pseudogenes, and one plastid-derived tRNA (supplementary table S3, Supplementary Material online).

Four ORFs (937 [Q903MT_gene558], 507 [Q903MT_gene3172], 371 [Q903MT_gene4186], and 221 [Q903MT_gene4187] amino acids) contain an organellar DNA polymerase type B (DNA_pol_B_2) Pfam family (El-Gebali et al. 2019). The largest one also contains a segment homologous to a putative cell-wall binding lipoprotein (YkyA) Pfam domain. These ORFs have homology to DNA polymerase genes found in the mitochondria of *P. glauca* and the angiosperms *Cocos nucifera*, *Daucus carota*, *Helianthus annuus*, and *Silene vulgaris*. Two ORFs (781 [Q903MT_gene4185] and 560 [Q903MT_gene6929] amino acids) contain an RNA polymerase (RNA_pol) domain. These ORFs have homology to DNA-dependent RNA polymerase genes found in the mitochondria of *P. glauca* and the angiosperms *Beta vulgaris*, *C. nucifera*, *D. carota*, and *Phoenix dactylifera*. The two largest genes of the Sitka spruce mitochondrial genome are these putative DNA and RNA polymerase genes.

The *matR* gene [Q903MT_gene5018] and three additional ORFs (476 [Q903MT_gene679], 197 [Q903MT_gene3613], and 163 [Q903MT_gene5889] amino acids) contain a reverse transcriptase, or RNA-dependent DNA polymerase, (RT_like) NCBI conserved protein domain with similarity to the group II intron reverse transcriptase/maturase (group_II_RT_mat) NCBI conserved protein domain (Marchler-Bauer et al. 2017). We hypothesize that these ORFs may be additional maturases involved in splicing (Matsuura 2001). These ORFs have homology to mitochondrial genes of *P. glauca* and the angiosperm *Utricularia reniformis*.

## Conclusion

The 5.5-Mb mitochondrial genome of Sitka spruce is among the largest ones in plants, and is the largest complete mitochondrial genome reported to date for a gymnosperm. It follows the trend seen for spruce and conifer nuclear genomes, which are also among the largest in plants (De La Torre et al. 2014). The physical structure of the Sitka spruce mitochondrial genome is not the typical circularly mapping single chromosome, but multipartite. The larger component of the assembly graph exhibits a rosette-like structure, mirroring the rosette-like structures observed in electron micrographs of mitochondrial DNA (Backert and Börner 2000). The intricate structure and the discovery of large repeat elements suggest the presence of active sites for hypothesized recombination-dependent replication of the mitochondrial genome (Gualberto et al. 2014; Sullivan et al. 2020). Considering heteroplasmy resulting from naturally hybridizing species of spruce and paternal leakage of mitochondria, intermolecular recombination would result in interspecific hybridization of the mitochondrial genome, which has been reported to occur in natural spruce populations (Jaramillo-Correa and Bousquet 2005). The complete mitochondrial genome of Sitka spruce, a gymnosperm, should prove invaluable to future investigations into the genome structure and mechanism of replication of conifer mitochondrial genomes.

## Supplementary Material

evaa108_Supplementary_Data

## Notes

**Data deposition:** The Oxford Nanopore MinION sequencing data are available at the SRA under accessions SRX5081713 and SRR8264752–SRR8264769. The Illumina HiSeq sequencing data have SRA accession SRR5028199. The Sitka spruce (*Picea sitchensis*) genotype Q903 complete mitochondrial genome is available at NCBI GenBank under accessions MK697696–MK697708.

## References

[evaa108-B1] Altschul SF, Gish W, Miller W, Myers EW, Lipman DJ. 1990 Basic local alignment search tool. J Mol Biol. 215(3):403–410. 2231712 10.1016/S0022-2836(05)80360-2

[evaa108-B2] Backert S, Börner T. 2000 Phage T4-like intermediates of DNA replication and recombination in the mitochondria of the higher plant *Chenopodium album* (L.). Curr Genet. 37(5):304–314. 10853767 10.1007/s002940050532

[evaa108-B3] Backert S, Lynn Nielsen B, Börner T. 1997 The mystery of the rings: structure and replication of mitochondrial genomes from higher plants. Trends Plant Sci. 2(12):477–483.

[evaa108-B4] Birol I, et al 2013 Assembling the 20 Gb white spruce (*Picea glauca*) genome from whole-genome shotgun sequencing data. Bioinformatics 29(12):1492–1497. 23698863 10.1093/bioinformatics/btt178 PMC3673215

[evaa108-B5] Chaw S-M, et al 2008 The mitochondrial genome of the gymnosperm *Cycas taitungensis* contains a novel family of short interspersed elements, Bpu sequences, and abundant RNA editing sites. Mol Biol Evol. 25(3):603–615. 18192697 10.1093/molbev/msn009

[evaa108-B6] Coombe L, et al 2016 Assembly of the complete Sitka spruce chloroplast genome using 10X genomics’ GemCode sequencing data. PLoS One 11(9):e0163059. 27632164 10.1371/journal.pone.0163059 PMC5025161

[evaa108-B8] Cupp JD, Nielsen BL. 2014 Minireview: DNA replication in plant mitochondria. Mitochondrion 19:231–237. 24681310 10.1016/j.mito.2014.03.008 PMC4177014

[evaa108-B9] De La Torre AR, et al 2014 Insights into conifer giga-genomes. PLANT Physiol. 166(4):1724–1732. 25349325 10.1104/pp.114.248708 PMC4256843

[evaa108-B10] El-Gebali S, et al 2019 The Pfam protein families database in 2019. Nucleic Acids Res. 47(D1):D427–D432. 30357350 10.1093/nar/gky995 PMC6324024

[evaa108-B12] Gualberto JM, et al 2014 The plant mitochondrial genome: dynamics and maintenance. Biochimie 100:107–120. 24075874 10.1016/j.biochi.2013.09.016

[evaa108-B13] Guo W, et al 2016 Ginkgo and Welwitschia mitogenomes reveal extreme contrasts in gymnosperm mitochondrial evolution. Mol Biol Evol. 33(6):1448–1460. 26831941 10.1093/molbev/msw024

[evaa108-B14] Guo W, et al 2020 Extensive shifts from cis to trans splicing of gymnosperm mitochondrial introns. Mol Biol Evol. 37(6):1615–1620. 10.1093/molbev/msaa029 32027368

[evaa108-B15] Hiesel R, Wissinger B, Schuster W, Brennicke A. 1989 RNA editing in plant mitochondria. Science 246(4937):1632–1634. 2480644 10.1126/science.2480644

[evaa108-B16] Holt C, Yandell M. 2011 MAKER2: an annotation pipeline and genome-database management tool for second-generation genome projects. BMC Bioinformatics 12(1):491. 10.1186/1471-2105-12-491 PMC3280279 22192575

[evaa108-B17] Hyatt D, et al 2010 Prodigal: prokaryotic gene recognition and translation initiation site identification. BMC Bioinformatics 11(1):119. 10.1186/1471-2105-11-119 PMC2848648 20211023

[evaa108-B18] Jackman SD, et al 2016 Organellar genomes of White spruce (*Picea glauca*): assembly and annotation. Genome Biol Evol. 8(1):29–41. 10.1093/gbe/evv244 PMC4758241 26645680

[evaa108-B19] Jaramillo-Correa JP, Bousquet J. 2005 Mitochondrial genome recombination in the zone of contact between two hybridizing conifers. Genetics 171(4):1951–1962. 16118197 10.1534/genetics.105.042770 PMC1456118

[evaa108-B20] Kalvari I, et al 2018 Rfam 13.0: shifting to a genome-centric resource for non-coding RNA families. Nucleic Acids Res. 46(D1):D335–D342. 29112718 10.1093/nar/gkx1038 PMC5753348

[evaa108-B21] Kamikawa R, Shiratori T, Ishida K-I, Miyashita H, Roger AJ. 2016 Group II intron-mediated trans-splicing in the gene-rich mitochondrial genome of an enigmatic eukaryote, *Diphylleia rotans*. Genome Biol Evol. 8(2):458–466. 26833505 10.1093/gbe/evw011 PMC4779616

[evaa108-B22] Kan SL, et al 2020 The complete mitochondrial genome of *Taxus cuspidata* (*Taxaceae*): eight protein-coding genes have transferred to the nuclear genome. BMC Evol Biol. 20(1):10. 31959109 10.1186/s12862-020-1582-1 PMC6971862

[evaa108-B23] Knoop V, Schuster W, Wissinger B, Brennicke A. 1991 Trans splicing integrates an exon of 22 nucleotides into the nad5 mRNA in higher plant mitochondria. EMBO J. 10(11):3483–3493. 1915303 10.1002/j.1460-2075.1991.tb04912.x PMC453077

[evaa108-B24] Kolmogorov M, Yuan J, Lin Y, Pevzner P. 2019 Assembly of long error-prone reads using repeat graphs. Nat Biotechnol. 37(5):540–546. 30936562 10.1038/s41587-019-0072-8

[evaa108-B25] Kovar L, et al 2018 PacBio-based mitochondrial genome assembly of *Leucaena trichandra* (*Leguminosae*) and an intrageneric assessment of mitochondrial RNA editing. Genome Biol Evol. 10(9):2501–2517. 30137422 10.1093/gbe/evy179 PMC6161758

[evaa108-B26] Kozik A, et al 2019 The alternative reality of plant mitochondrial DNA: one ring does not rule them all. PLoS Genet. 15(8):e1008373. 31469821 10.1371/journal.pgen.1008373 PMC6742443

[evaa108-B27] Lagesen K, et al 2007 RNAmmer: consistent and rapid annotation of ribosomal RNA genes. Nucleic Acids Res. 35(9):3100–3108. 17452365 10.1093/nar/gkm160 PMC1888812

[evaa108-B28] Lambowitz AM, Zimmerly S. 2011 Group II introns: mobile ribozymes that invade DNA. Cold Spring Harb Persp Biol. 3(8):a003616. 10.1101/cshperspect.a003616 PMC3140690 20463000

[evaa108-B29] Lang BF, Laforest M-J, Burger G. 2007 Mitochondrial introns: a critical view. Trends Genet. 23(3):119–125. 17280737 10.1016/j.tig.2007.01.006

[evaa108-B30] Laslett D. 2004 ARAGORN, a program to detect tRNA genes and tmRNA genes in nucleotide sequences. Nucleic Acids Res. 32(1):11–16. 14704338 10.1093/nar/gkh152 PMC373265

[evaa108-B31] Li H. 2016 Minimap and miniasm: fast mapping and de novo assembly for noisy long sequences. Bioinformatics 32(14):2103–2110. 27153593 10.1093/bioinformatics/btw152 PMC4937194

[evaa108-B32] Li H. 2018 Minimap2: pairwise alignment for nucleotide sequences. Bioinformatics 34(18):3094–3100. 29750242 10.1093/bioinformatics/bty191 PMC6137996

[evaa108-B33] Lowe TM, Eddy SR. 1997 tRNAscan-SE: a program for improved detection of transfer RNA genes in genomic sequence. Nucleic Acids Res. 25(5):955–964. 9023104 10.1093/nar/25.5.955 PMC146525

[evaa108-B34] Marchler-Bauer A, et al 2017 CDD/SPARCLE: functional classification of proteins via subfamily domain architectures. Nucleic Acids Res. 45(D1):D200–D203. 27899674 10.1093/nar/gkw1129 PMC5210587

[evaa108-B35] Maréchal A, Brisson N. 2010 Recombination and the maintenance of plant organelle genome stability. New Phytol. 186(2):299–317. 20180912 10.1111/j.1469-8137.2010.03195.x

[evaa108-B36] Matsuura M. 2001 Mechanism of maturase-promoted group II intron splicing. EMBO J. 20(24):7259–7270. 11743002 10.1093/emboj/20.24.7259 PMC125332

[evaa108-B37] Mower JP, Sloan DB, Alverson AJ. 2012 Plant mitochondrial genome diversity: the genomics revolution. Plant Genome Divers. 1:123–144.

[evaa108-B38] Nawrocki EP, Kolbe DL, Eddy SR. 2009 Infernal 1.0: inference of RNA alignments. Bioinformatics 25(10):1335–1337. 19307242 10.1093/bioinformatics/btp157 PMC2732312

[evaa108-B39] Nystedt B, et al 2013 The Norway spruce genome sequence and conifer genome evolution. Nature 497(7451):579–584. 23698360 10.1038/nature12211

[evaa108-B40] Oldenburg DJ, Bendich AJ. 2015 DNA maintenance in plastids and mitochondria of plants. Front Plant Sci. 6:10.3389/fpls.2015.00883 PMC4624840 26579143

[evaa108-B41] Sakamoto W, Tan S-H, Murata M, Motoyoshi F. 1997 An unusual mitochondrial atp9-rpl16 cotranscript found in the maternal distorted leaf mutant of *Arabidopsis thaliana*: implication of GUG as an initiation codon in plant mitochondria. Plant Cell Physiol. 38(8):975–979. 9327595 10.1093/oxfordjournals.pcp.a029261

[evaa108-B42] Seemann T. 2014 Prokka: rapid prokaryotic genome annotation. Bioinformatics 30(14):2068–2069. 24642063 10.1093/bioinformatics/btu153

[evaa108-B43] Skippington E, Barkman TJ, Rice DW, Palmer JD. 2015 Miniaturized mitogenome of the parasitic plant *Viscum scurruloideum* is extremely divergent and dynamic and has lost all nad genes. Proc Natl Acad Sci USA. 112(27):E3515–24. 26100885 10.1073/pnas.1504491112 PMC4500244

[evaa108-B44] Slater G, Birney E. 2005 Automated generation of heuristics for biological sequence comparison. BMC Bioinformatics 6(1):31. 15713233 10.1186/1471-2105-6-31 PMC553969

[evaa108-B45] Sloan DB, et al 2012 Rapid evolution of enormous, multichromosomal genomes in flowering plant mitochondria with exceptionally high mutation rates. PLoS Biol. 10(1):e1001241. 22272183 10.1371/journal.pbio.1001241 PMC3260318

[evaa108-B46] Sullivan AR, et al 2020 The mitogenome of Norway Spruce and a reappraisal of mitochondrial recombination in plants. Genome Biol Evol. 12(1):3586–3598. 31774499 10.1093/gbe/evz263 PMC6944214

[evaa108-B47] Vaser R, Sović I, Nagarajan N, Šikić M. 2017 Fast and accurate de novo genome assembly from long uncorrected reads. Genome Res. 27(5):737–746. 28100585 10.1101/gr.214270.116 PMC5411768

[evaa108-B48] Vlcek C, Marande W, Teijeiro S, Lukeš J, Burger G. 2011 Systematically fragmented genes in a multipartite mitochondrial genome. Nucleic Acids Res. 39(3):979–988. 20935050 10.1093/nar/gkq883 PMC3035467

[evaa108-B49] Walker BJ, et al 2014 Pilon: an integrated tool for comprehensive microbial variant detection and genome assembly improvement. PLoS One 9(11):e112963. 25409509 10.1371/journal.pone.0112963 PMC4237348

[evaa108-B50] Wang W, et al 2018 Assembly of chloroplast genomes with long- and short-read data: a comparison of approaches using *Eucalyptus pauciflora* as a test case. BMC Genomics 19(1). 10.1186/s12864-018-5348-8 PMC6311037 30594129

[evaa108-B51] Warren RL, et al 2015 Improved white spruce (*Picea glauca*) genome assemblies and annotation of large gene families of conifer terpenoid and phenolic defense metabolism. Plant J. 83(2):189–212. 26017574 10.1111/tpj.12886

[evaa108-B52] Wick RR, Judd LM, Gorrie CL, Holt KE. 2017a. Completing bacterial genome assemblies with multiplex MinION sequencing. Microb Genomics. 3(10):e000132. 10.1099/mgen.0.000132 PMC5695209 29177090

[evaa108-B53] Wick RR, Judd LM, Gorrie CL, Holt KE. 2017b. Unicycler: resolving bacterial genome assemblies from short and long sequencing reads. PLOS Comput Biol. 13(6):e1005595. 28594827 10.1371/journal.pcbi.1005595 PMC5481147

[evaa108-B54] Wick RR, Schultz MB, Zobel J, Holt KE. 2015 Bandage: interactive visualization of de novo genome assemblies. Bioinformatics 31(20):3350–3352. 26099265 10.1093/bioinformatics/btv383 PMC4595904

