# Supplementary material for: Complete Mitochondrial Genome of a Gymnosperm, Sitka Spruce (Picea sitchensis), Indicates a Complex Physical Structure
Source: Genome Biol Evol. 2020 May 25;12(7):1174–9. doi: 10.1093/gbe/evaa108 (PMC7486957; doi:10.1093/gbe/evaa108)
Supplement: evaa108_Supplementary_Data [file evaa108_supplementary_data.docx]

Supplementary material for manuscript “Complete mitochondrial genome of a gymnosperm, Sitka spruce (*Picea sitchensis*), indicates a complex physical structure” by S. Jackman *et al*.

**Supplementary Methods**

**Assembly**

We selected putative mitochondrial reads by aligning the Nanopore reads to our initial miniasm+racon nanopore read assembly using Minimap2 (Li 2018), and retained reads with an alignment score of 5000 or more (Supplementary Figure S1). We assembled these reads using Unicycler (Wick et al. 2017b). This assembly yielded one circular contig and many linear contigs with no adjacent contigs, indicating that the assembly may not yet be complete, unless the genome was composed of linear chromosomes. We repeated the alignment of the Nanopore reads, this time to the Unicycler assembly, and again retained reads with an alignment score of 5000 or more. We assembled these reads using Flye (Kolmogorov et al. 2018), taking the output assembly graph that identifies repeats that are longer than the read length and determines their precise boundaries. The resulting Flye assembly was polished using Racon. Contigs with homology to the white spruce mitochondrion were selected using Bandage, which uses BLASTN, and required an alignment length of at least 5 kbp and sequence identity of 90% or more. Unambiguous adjacent contigs were merged using the Bandage operation “Merge all possible nodes”.

In addition to having a generally high sequencing error rate, nanopore reads from current leading technologies (Oxford Nanopore Technologies) do not represent the length of homopolymer repeats accurately. To correct for sequencing and homopolymer length errors, we polished the assembly using one flow cell of Illumina HiSeq sequencing reads of the same DNA extraction, yielding 59-fold depth of coverage of the mitochondrial genome. We used Unicycler Polish to iteratively align the reads to the assembly using Bowtie2 (Langmead & Salzberg 2012), and correct the consensus sequence using Pilon (Walker et al. 2014). This iterative polishing process yielded no further corrections on the tenth round. Unicycler Polish applies Assembly Likelihood Estimate (ALE) (Clark et al. 2013) to each round to verify that the assembly of the final round of polishing resembles the reads the most. While annotating the genome, we found five indel errors in homopolymer runs that disrupted the reading frame of a gene. These five indel errors were corrected manually after inspecting the sequencing data.

**Annotation**

Following automated annotation, we reviewed coding genes for completeness, compared to their best BLASTP match, and corrected the annotation, most often for aspects that are particular to plant mitochondria. We manually corrected the annotation of genes to address start codons created by RNA editing of ACG to the start codon AUG, and editing of GCG to the alternative start codon GUG (see results for details). Three genes display atypical start codons: *rpl16* uses a GUG start codon (Sakamoto et al. 1997); *rps19* uses a GUG start codon created by RNA editing GCG, seen also in *Pinus strobus* AJP33554.1; *matR* appears to use an unusual GGG start codon, seen also in *Cycas taitungensis* YP_001661429.1 (Chaw et al. 2008) and *Pinus strobus* AJP33535.1. The gene *sdh4* was missed by automatic annotation, as its coding sequence was found to overlap with *cox3* by 73 bp on the same strand.

We reviewed splice sites, and adjusted their position to agree with the expected splicing motifs of group II introns when possible. We confirmed the presence of domain V of the group II intron upstream of the 3’ splice site, identified by RNAweasel or Infernal. We manually annotated trans-spliced introns by comparing alignments of homologous proteins to the genome. We determined the 5’ and 3’ splice sites similarly to cis-spliced introns, looking for expected group II splicing motifs, and domain V upstream of the 3’ splice site. When Infernal did find a match to RFAM Intron_gpII (RF00029), it frequently identified the precise 3’ splice site, in agreement with protein sequence homology.

The scripts to assemble and annotate the Sitka spruce mitochondrial genome are available online at <https://github.com/sjackman/psitchensismt>.

**Supplementary results**

The full complement of rRNA genes are present in Sitka spruce, shown in Supplementary Table 1. Unlike rRNA genes of other gymnosperms, the Sitka spruce rRNA genes are present in multiple copies. The 5S rRNA gene *rrn5* is present in four copies. The small subunit rRNA gene *rrn18* is present in three copies, though one copy is found on the 27 kbp repeat segment with an estimated copy number of two. One copy of the large subunit rRNA gene *rrn26* is present, though it is found on the 24 kbp repeat segment, which also has an estimated copy number of two.

Sitka spruce has 27 tRNA genes, representing 18 distinct anticodons, coding for 15 distinct amino acids, DEHIKLMNPQRTVWY (Supplementary Table 2). tRNA genes coding for the amino acids ACFGS are absent in Sitka spruce, and also absent in *Welwitschia*. *trnM-CAU* exhibits six copies, *trnD-GUC* three copies, and *trnY-GUA* two copies. All other tRNA genes are single copy. *trnN-GUU*, *trnV-UAC*, and one copy of *trnfM-CAU* are derived from plastid origins. One cis-spliced intron is observed in the plastid-derived *trnV-UAC* gene, also seen in *Cycas taitungensis*. Six tRNA genes (*trnL-CAA*, *trnR-CCG*, *trnR-GCG*, *trnT-AGU*, *trnT-UGU*, and *trnY-AUA*) found in Sitka spruce are absent in *Cycas*, *Ginkgo*, and *Welwitschia*.

In addition to three plastid-derived tRNA genes, ten partial plastid genes are found in the 14 kbp of plastid-derived sequence: *atpB*, *atpE*, *atpF*, *chlN*, *petA*, *psaA*, *rps3*, *rrn18*, a partial copy of *rpl2*, and a partial *trnS-GGA* gene with homology to *Cycas taitungensis*. The *rpl2* partial gene is more similar to eudicot plastids (77% identical to *Helwingia himalaica*, *Robinia pseudoacacia*, and many other eudicots) than it is to the Sitka spruce plastid (66% identical).

## Introns

Although the same 27 introns are found in the same 11 genes as *Cycas taitungensis* (Chaw et al. 2008; Guo et al. 2016), eight introns that are cis-spliced in *Cycas* are trans-spliced in Sitka spruce, more than doubling the number of trans-spliced introns found in *Cycas*. Nearly half of the introns in Sitka spruce are trans-spliced. All introns are group II introns, whose domain V was identified by both RNAweasel and Infernal, with one exception. The first intron of *nad1* is trans-spliced in Sitka spruce and other gymnosperms (Guo et al. 2016). No domain V is detectable by Infernal, either downstream of exon 1 or upstream of exon 2 in Sitka spruce nor any of *Cycas*, *Ginkgo*, and *Welwitschia*. The genomic disruption of this intron may occur in domain V itself, as is seen in *cox2* of *Diphylleia rotans* (Kamikawa et al. 2016).

The fourth intron of *nad1* is cis-spliced, and it contains *matR* in *Cycas*, trans-spliced with a single disruption in Sitka spruce, and trans-spliced with two distinct genomic disruptions in *Welwitschia mirabilis* (Figure S2 of Guo et al. 2016). Whereas *matR* is found in a cis-spliced intron in *Cycas* and free-standing in *Welwitschia*, it is found upstream of *nad1* exon 5 in Sitka spruce. In this regard, Sitka spruce appears to be an evolutionary midpoint between *Cycas* and *Welwitschia*. Sitka spruce has not however experienced the extensive gene loss observed in *Welwitschia*.

A second partial copy of *nad5* is found in Sitka spruce with one cis-spliced group II intron, representing exons 4 and 5. The translated protein sequence of this partial gene is more similar however to eudicot mitochondria (99% identical to both *Chrysobalanus icaco* and *Hirtella racemosa*, >95% identical to many other eudicots, and 94% identical to one monocot *Triantha glutinosa*) than to the complete *nad5* of Sitka spruce (76% identical). It may have been acquired by horizontal gene transfer, as is frequently reported in plant mitochondria (Richardson & Palmer 2006) of both gymnosperms (Won & Renner 2003) and angiosperms (Bergthorsson et al. 2003). This interpretation of horizontal gene transfer in plant mitochondria is not universally accepted (Goremykin et al. 2008). This partial copy of *nad5* is also found in white spruce (Jackman et al. 2015) with 100% nucleotide identity. This level of conservation between Sitka spruce, white spruce, and angiosperms suggests that this partial gene may be functional. We find no upstream domain V, whose presence would indicate that it may be part of a larger trans-spliced gene. A putative alternative GUG start codon created by RNA editing of GCG could initiate translation of this partial gene.

RNAweasel identifies 34 group II domain V regions in Sitka spruce, 26 of which are associated with the intron of a gene. Two domain V regions are found in the cis-spliced introns of the pseudogenes *Ψnad5* and plastid-derived *Ψrpl2*. The remaining six domain V regions are not associated with a gene, and further investigation would be needed to determine whether they may also be partial fragments of pseudogene introns.

The splice-site motifs of the 14 cis-spliced genes of the Sitka spruce mitochondrial genome are shown in Supplementary Figure S2, visualized by WebLogo (Crooks 2004). Because its position is variable, the bulged adenosine of the 3’ splice site, typically found at position -7 or -8, is not readily apparent.

Trans-splicing is a frequently-employed mechanism of plant mitochondria to compensate for genomic structural instability, and Sitka spruce has a record number of trans-spliced introns (13 trans-spliced introns observed in 10 protein-coding genes).

**Supplementary tables**

**Supplementary Table 1**. rRNA gene content of four gymnosperms. This table is adapted from Table S1 of Guo et al. (2016) with the addition of Sitka spruce. *One copy is present on a repeat segment with an estimated copy number of two.

| Gene | Cycas | Ginkgo | Sitka | Welwitschia |
| --- | --- | --- | --- | --- |
| rrn5 | 1 | 1 | 4 | 1 |
| rrn18 | 1 | 1 | 3* | 1 |
| rrn26 | 1 | 1 | 1* | 1 |
| Total rRNA | 3 | 3 | 8 | 3 |

**Supplementary Table 2**. tRNA content of four gymnosperms. Sitka spruce has 27 tRNA genes, one of which is cis-spliced, with 18 distinct anticodons, coding for 15 distinct amino acids. This table is adapted from Table S1 of Guo et al. (2016) with the addition of Sitka spruce. (i) Contains a cis-spliced group II intron. *Anticodon is inferred to be edited (Weber et al. 1990).

| Gene | Cycas | Ginkgo | Sitka | Welwitschia |
| --- | --- | --- | --- | --- |
| trnC-GCA | 1 | 1 | - | - |
| trnD-GUC | 1 | 1 | 3 | 1 |
| trnE-UUC | 1 | 1 | 1 | 1 |
| trnF-GAA | 1 | 2 | - | - |
| trnG-GCC | 1 | - | - | - |
| trnG-UCC | - | 1* | - | - |
| trnH-GUG | 1 | 1 | 1 | - |
| trnI-CAU | 1* | 1* | 1* | 1* |
| trnK-UUU | 1 | 1* | 1 | - |
| trnL-CAA | - | - | 1 | - |
| trnL-UAA | 1* | 2 | - | - |
| trnL-UAG | 1 | 1 | - | - |
| trnM-CAU | 6 | 2 | 6 | 1 |
| trnN-GUU | 1 | - | 1 | - |
| trnP-AGG | 1 | 1 | 1 | - |
| trnP-UGG | 1 | 1 | 1 | - |
| trnQ-UUG | 1* | 1 | 1 | 1 |
| trnR-ACG | - | - | - | 1 |
| trnR-CCG | - | - | 1 | - |
| trnR-GCG | - | - | 1 | - |
| trnR-UCU | 1* | 1 | - | - |
| trnS-GCU | 1 | 1 | - | - |
| trnS-GGA | 1 | - | - | - |
| trnS-UGA | 1 | 1 | - | - |
| trnT-AGU | - | - | 1 | - |
| trnT-UGU | - | - | 1 | - |
| trnV-UAC (i) | 1 | - | 1 | - |
| trnW-CCA | 1 | 2 | 1 | 1 |
| trnY-AUA | - | - | 1 | - |
| trnY-GUA | 1 | 1 | 2 | 1 |
| Total tRNA | 27 | 23 | 27 | 8 |

**Supplementary Table 3**. Intron content of four gymnosperms. Sitka spruce has 29 introns, 16 cis-spliced (•) and 13 trans-spliced (T), in ten protein coding genes, two pseudogenes (Ψ), and one tRNA. “T²” indicates a tripartite (double trans-spliced) intron. “-” indicates intron absence. “x” indicates gene absence. “cp” indicates plastid-derived. This table is adapted from Guo et al. (2016) with the addition of Sitka spruce.

| Gene | Intron | Cycas | Ginkgo | Sitka | Welwitschia |
| --- | --- | --- | --- | --- | --- |
| ccmFc | 1 | • | • | • | - |
| cox2 | 1 | • | • | T | - |
|  | 2 | • | • | T | T |
| nad1 | 1 | T | T | T | T |
|  | 2 | • | • | • | - |
|  | 3 | T | T | T | T |
|  | 4 | • | • | T | T² |
| nad2 | 1 | • | • | • | - |
|  | 2 | T | T | T | T |
|  | 3 | • | • | • | - |
|  | 4 | • | • | T | - |
| nad4 | 1 | • | • | T | • |
|  | 2 | • | • | • | - |
|  | 3 | • | • | • | - |
| nad5 | 1 | • | • | • | - |
|  | 2 | T | T | T | T |
|  | 3 | T | T | T | T |
|  | 4 | • | • | • | - |
| nad7 | 1 | • | • | • | - |
|  | 2 | • | • | T | • |
|  | 3 | • | • | • | - |
|  | 4 | • | • | T | • |
| rpl2 | 1 | • | • | T | x |
| rps3 | 1 | • | • | • | - |
|  | 2 | • | • | • | - |
| rps10 | 1 | • | - | • | x |
| trnV-UAC (cp) | 1 | • | x | • | x |
| Ψnad5 | 4 | x | x | • | x |
| Ψrpl2 (cp) | 1 | x | x | • | x |
| Total cis (•) |  | 22 | 20 | 16 | 3 |
| Total trans (T) |  | 5 | 5 | 13 | 7 |
| Total introns |  | 27 | 25 | 29 | 10 |

**Supplementary figures**

**
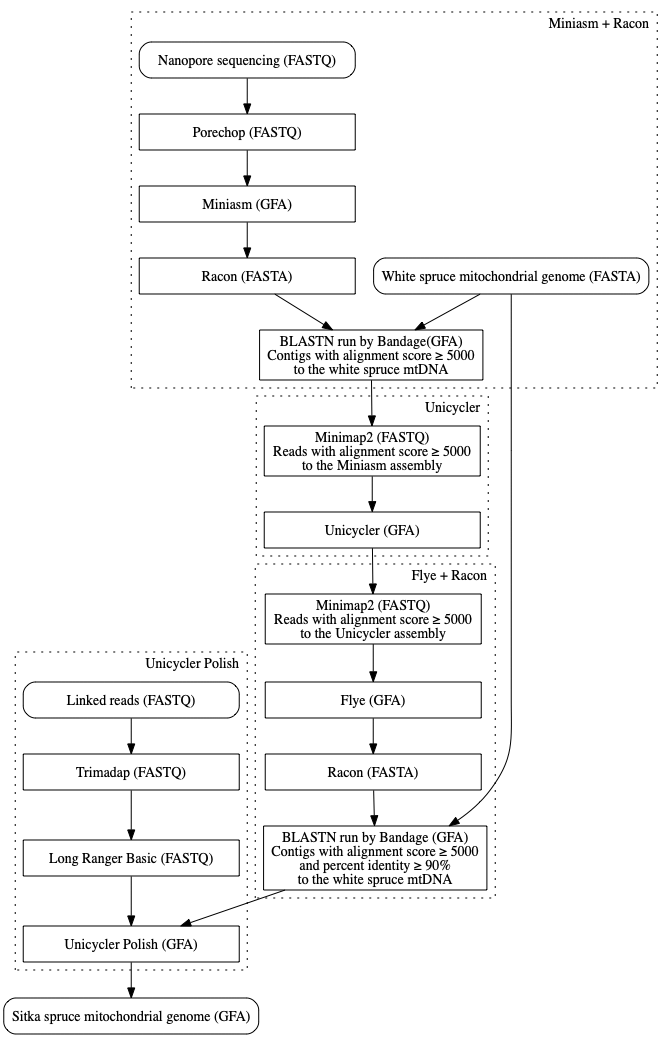
**

**Supplementary Fig. S1. Sitka spruce mitochondrion assembly pipeline.** Sitka spruce nanopore long reads were trimmed with Porechop (Wick et al. 2017a) and assembled with Miniasm (Li 2016). Contigs with homology to the white spruce (genotype PG29) mitochondrial genome (Jackman et al. 2015) were identified using Bandage (Wick et al. 2015). Nanopore reads were aligned back to our draft Sitka spruce assembly with minimap2 (Li 2016), segregated and assembled *de novo* with Unicycler (Wick et al. 2017b), and then with Flye (Kolmogorov et al. 2018). We polished the genome sequence assembly with Racon (Vaser et al. 2017) and Pilon (Walker et al. 2014) using Illumina HiSeq sequencing reads of the same DNA extraction.


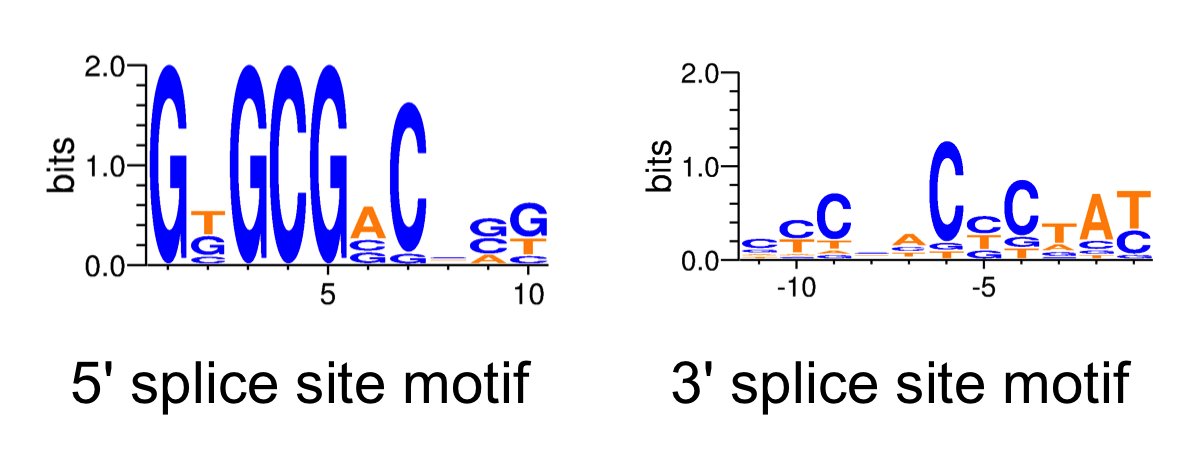
**Supplementary Fig. S2. The 5’ and 3’ splice-site motifs of the 14 cis-spliced genes of Sitka spruce.**

**Additional references**

Bergthorsson U, Adams KL, Thomason B, Palmer JD. 2003. Widespread horizontal transfer of mitochondrial genes in flowering plants. Nature. 424:197–201.

Clark SC, Egan R, Frazier PI, Wang Z. 2013. ALE: a generic assembly likelihood evaluation framework for assessing the accuracy of genome and metagenome assemblies. Bioinformatics. 29:435–443.

Goremykin VV, Salamini F, Velasco R, Viola R. 2008. Mitochondrial DNA of Vitis vinifera and the Issue of Rampant Horizontal Gene Transfer. Molecular Biology and Evolution. 26:99–110.

Langmead B, Salzberg SL. 2012. Fast gapped-read alignment with Bowtie 2. Nature Methods. 9:357–359.

Richardson AO, Palmer JD. 2006. Horizontal gene transfer in plants. Journal of Experimental Botany. 58:1–9.

Weber F, Dietrich A, Weil J-H, Maréchal-Drouard L. 1990. A potato mitochondrial isoleucine tRNA is coded for by a mitochondrial gene possessing a methionine anticodon. Nucleic Acids Research. 18:5027–5030.

Won H, Renner SS. 2003. Horizontal gene transfer from flowering plants to Gnetum. Proceedings of the National Academy of Sciences. 100:10824–10829.
